# Portable Freehand 3D Breast Ultrasound Using a Dual-Rotary-Encoder 2DoF Tracking Framework

**DOI:** 10.3390/s26134080

**Published:** 2026-06-27

**Authors:** Syahid Al Irfan, Oky Dicky Ardiansyah Prima

**Affiliations:** Graduate School of Software and Information Science, Iwate Prefectural University, 152-52, Takizawa-shi 020-0693, Iwate, Japan; prima@iwate-pu.ac.jp

**Keywords:** freehand 3D ultrasound, breast imaging, pose estimation, rotary encoder, volumetric reconstruction, portable ultrasound

## Abstract

Freehand three-dimensional (3D) ultrasound enables cost-effective volumetric breast imaging, but accurate reconstruction requires reliable probe tracking during manual scanning. This study proposes a portable freehand 3D ultrasound framework using dual-rotary-encoder two-degree-of-freedom (2DoF) pose sensing to measure probe displacement and inclination during breast scanning. A slip-resistant roller mechanism and time-aware trajectory modeling were introduced to improve measurement robustness under practical scanning conditions. The framework was evaluated through robotic experiments and phantom-based volumetric reconstruction. Positional displacement experiments achieved root mean square errors (RMSEs) of 0.38 mm on dry surfaces and 0.81 mm under gel-coated conditions. Inclination sensing using the rotary encoder outperformed an inertial measurement unit (IMU), achieving an RMSE of 2.76° with improved temporal stability. Reconstruction experiments using a breast phantom with spherical inclusions demonstrated successful volumetric visualization across multiple scanning trajectories. Statistical analysis revealed significant effects of inclusion size and scanning trajectory on relative reconstruction error, as well as a significant interaction between the two factors. Larger inclusions generally exhibited lower relative errors, while the influence of scanning trajectory depended on the target size. These findings support the feasibility of the proposed reduced-dimensional mechanical pose sensing approach for reliable freehand 3D ultrasound reconstruction with reduced hardware complexity.

## 1. Introduction

Breast cancer remains a major global public health concern and is one of the leading causes of cancer-related mortality among women. According to the World Cancer Research Fund, more than 2.3 million new cases are diagnosed annually, highlighting the critical importance of early detection for improving long-term survival outcomes [1].

Currently, mammography is the gold-standard screening modality and has demonstrated high efficacy in detecting early-stage malignancies in women with fatty breast tissue. Sensitivity in this population is typically in the range of 75–85% [2]. However, in women with dense breast tissue—a demographic encompassing 40–50% of the screening population—mammography’s effectiveness drops significantly. Both normal fibroglandular tissue and tumors appear radiopaque on X-ray images, resulting in a “masking effect” that reduces lesion conspicuity and increases the likelihood of false negatives. Sensitivity in such cases can fall to 60–70%, compared to 90% in non-dense breast [3,4].

To mitigate these limitations, adjunctive ultrasound imaging has been widely studied and adopted. A meta-analysis of 29 studies revealed that adding ultrasound to mammography increased the overall cancer detection rate by approximately 40%, uncovering an additional 3.8 cancers per 1000 women with negative mammograms [5]. In patients with dense breasts, the combined use of ultrasound and mammography improved sensitivity from 62% to 81%, with a specificity ranging between 82% and 87% [6]. Despite the demonstrated improvement in cancer detection when ultrasound is used as an adjunct screening modality, its clinical performance remains limited by substantial operator dependence during image acquisition and interpretation [7]. Variability in scanning technique and reader experience can affect diagnostic consistency and reproducibility across examinations. To address these limitations, three-dimensional (3D) ultrasound imaging has been developed, enabling the reconstruction of conventional two-dimensional (2D) ultrasound images into volumetric datasets [8]. By providing comprehensive spatial information and reducing reliance on operator technique, 3D ultrasound offers the potential for more standardized and reproducible breast imaging assessments.

A key observation is that surface-constrained breast scanning is dominated by translational motion along the skin and limited probe inclination. This suggests that accurate volumetric reconstruction in surface-constrained breast scanning may be achievable using a reduced-dimensional pose representation rather than full six-degree-of-freedom (6DoF) tracking. However, empirical validation of reduced-dimension pose modeling remains limited, and practical sensing frameworks that exploit this task-specific motion structure have not been sufficiently investigated.

Motivated by this gap, this study proposes a portable freehand 3D ultrasound framework based on a sensing mechanism using a dual-rotary-encoder that measures probe displacement and inclination as a two-degree-of-freedom (2DoF) pose representation. A slip-resistant roller mechanism was designed to preserve translational accuracy under gel-mediated contact, while a hinge-mounted rotary encoder provides stable inclination sensing. The framework further incorporates time-aware trajectory modeling and outlier handling to mitigate motion-induced reconstruction artifacts during manual scanning.

The proposed system was evaluated through controlled robotic experiments and phantom-based volumetric reconstruction. Results demonstrate millimeter-level positional accuracy, improved inclination stability compared with IMU-based sensing, and consistent reconstruction across multiple scanning trajectories. Statistical analysis revealed significant effects of both inclusion size and scanning trajectory on reconstruction accuracy, together with a significant interaction between these factors, indicating that the influence of scanning trajectory depends on lesion size.

The main contributions of this study are as follows:

(1) The introduction of a dual-rotary-encoder probe sensing mechanism enabling reduced two-degree-of-freedom pose estimation for freehand ultrasound;
(2) The design of a slip-resistant translational measurement strategy suitable for gel-based clinical scanning;
(3) Empirical validation demonstrating that practical volumetric breast ultrasound reconstruction can be achieved using reduced-dimensional pose sensing under constrained scanning conditions;
(4) A portable framework supporting practical point-of-care breast imaging and longitudinal lesion monitoring.

## 2. Literature Review

3D ultrasound technologies for breast cancer screening have progressed rapidly in response to limitations of traditional 2D ultrasound and mammography, particularly in patients with dense breast tissue. These technologies can be grouped into five main categories: HHUS, Automated Breast Ultrasound Systems (ABUS), Freehand Ultrasound with External Tracking (FWET), Autonomous Robotic and Actuator-Based Systems (ARABS), and AI-Assisted 3D Ultrasound Reconstruction (AI-3DUR). Each approach offers distinct advantages while presenting specific technical and practical challenges related to reproducibility, hardware complexity, and portability.

### 2.1. Handheld 2D Ultrasound

HHUS remains the most widely used supplemental imaging modality due to its accessibility and real-time imaging capability. Advances in transducer design, beamforming techniques, and image processing have improved lesion visualization and tissue characterization. For example, high-frequency linear transducers enable finer delineation of tissue boundaries [9], while Doppler imaging and elastography provide additional functional information for lesion assessment [10].

Despite these improvements, HHUS remains highly operator dependent and lacks volumetric context, limiting reproducibility and longitudinal assessment. Performance metrics such as sensitivity, specificity, lesion detection rate, and inter-observer variability highlight these challenges. Sensitivity in dense breast tissue typically ranges from 70% to 85%, but specificity is often reduced due to increased false positives [11]. These limitations have driven the development of volumetric ultrasound approaches.

### 2.2. Automated Breast Ultrasound Systems

ABUSs automate probe motion to acquire standardized 3D breast volumes. Typically mounted on fixed frames, these systems translate the transducer across the breast in controlled trajectories, improving reproducibility and reducing operator dependency. ABUSs have demonstrated increased cancer detection rates when used alongside mammography, particularly in dense breast populations [12,13].

Prototype systems such as the portable ABUS proposed by Park et al. achieved sub-millimeter trajectory accuracy and reconstruction errors below 3% [14]. Comparative studies indicate that ABUSs can reduce radiologist workload while maintaining diagnostic performance [15]. Recent systems integrate machine learning–based lesion detection to further enhance efficiency. However, ABUSs remain relatively expensive, bulky, and dependent on dedicated workstations, limiting widespread adoption in point-of-care environments.

### 2.3. Freehand Ultrasound with External Tracking

FWET integrates conventional probes with external tracking technologies to enable volumetric image reconstruction. These systems typically rely on optical tracking (e.g., structured light or marker-based motion capture) or mechanical rotary encoders that monitor the probe’s spatial trajectory.

Meza et al. [16] used structured-light scanning combined with optical markers to create 3D point clouds from breast phantom scans, achieving a mean error of less than 0.7 mm. Similarly, Jerald et al. [17] proposed a simplified linear track setup over water-filled phantoms to guide probe motion, although their system struggled with uneven surfaces.

The major advantage of this approach lies in its adaptability to existing ultrasound systems. However, calibration complexity, sensitivity to environmental lighting, and user training requirements remain significant barriers.

### 2.4. Autonomous Robotic and Actuator-Based Systems

ARABSs use robotic arms or actuators to guide probe motion based on preprogrammed or sensor-informed trajectories. These systems are designed to minimize human error and standardize data acquisition across users and settings.

Chen et al. [18] introduced a co-robotic arm platform that performed volumetric scanning guided by AR marker detection. Their system achieved accurate volumetric reconstructions and showed a lateral deviation error of only 1.58 mm when compared to ground truth data. Robotic systems also have the advantage of controlled pressure and trajectory, which can enhance tissue visualization consistency.

Despite their advantages, such systems are typically expensive, bulky, and require complex mechanical integration, limiting their application to research or high-resource clinical settings.

### 2.5. AI-Assisted 3D Ultrasound Reconstruction

AI-3DUR has emerged as a transformative approach to improve accuracy, reduce reliance on external tracking, and simplify the acquisition process. AI models—especially those based on convolutional neural networks (CNNs) and recurrent models like LSTMs—have been used for real-time pose estimation, motion correction, and volumetric image reconstruction.

Chen et al. [19] proposed the Sequence Prediction Reconstruction based on Acoustic Optical Localization, a deep-learning method using acoustic-optic data as pose input to reconstruct 3D volumes. Their system achieved an average pose estimation error of 1.55 mm per frame. Luo et al. [20] developed a system combining ultrasound with an IMU, using a deep learning model to address drift and estimate displacement, showing enhanced reconstruction accuracy over prior methods.

Recent studies have also investigated unsupervised learning methods and transformer-based models for image enhancement and spatial prediction [21,22]. While AI-based methods demonstrate strong potential for real-time, portable screening applications, they still require extensive training data and thorough validation across diverse clinical settings. Across these studies, common evaluation metrics include spatial reconstruction error (e.g., mean absolute error, root mean square error), volumetric consistency, CNR, and time efficiency (e.g., scan duration and interpretation time). Sensitivity and specificity—particularly in detecting small lesions within dense breast tissue—remain critical clinical performance indicators.

### 2.6. Synthesis and Research Gap

Across these categories, common evaluation metrics include spatial reconstruction error, volumetric consistency, contrast-to-noise ratio, acquisition time, and diagnostic performance indicators such as sensitivity and specificity. HHUS is limited by operator variability, ABUSs by cost and hardware constraints, FWET by calibration complexity, robotic systems by mechanical overhead, and AI-based approaches by data dependency. Table 1 summarizes the major 3D ultrasound approaches for breast cancer screening.

A key observation emerging from prior work is that probe motion requirements are task dependent. In surface-constrained breast scanning, motion is predominantly characterized by translation along the skin surface with limited angular variation. This suggests that full six-degree-of-freedom tracking may be unnecessary for reliable volumetric reconstruction in this specific clinical context. However, practical sensing frameworks that exploit this reduced motion structure remain underexplored, and empirical validation is limited.

Therefore, a critical gap exists in the development of compact, user-friendly 3D ultrasound systems that balance reconstruction accuracy, hardware simplicity, portability, and accessibility. Addressing this gap requires task-specific pose sensing approaches capable of reducing system complexity while maintaining volumetric fidelity. The present study investigates such an approach through a dual-rotary-encoder sensing framework that directly measures translational displacement and probe inclination as a reduced two-degree-of-freedom representation.

## 3. Materials and Methods

This study proposes a portable freehand 3D ultrasound acquisition framework that integrates mechanical pose sensing with conventional ultrasound imaging to enable volumetric breast reconstruction in resource-constrained settings. The methodological design is grounded in the hypothesis that breast surface scanning can be accurately represented using a reduced pose space, where probe translation and inclination constitute the dominant variables governing slice placement.

To operationalize this concept, a dual-rotary-encoder architecture was developed to provide 2DoF pose estimation, capturing probe displacement along the scanning path and angular deviation relative to the surface normal. This task-specific sensing strategy replaces conventional 6DoF tracking systems with a compact mechanical alternative while preserving reconstruction fidelity.

The methodological pipeline comprises three tightly coupled stages: a dual-rotary-encoder probe pose-sensing, synchronized acquisition of ultrasound frames with pose information, followed by standardized data integration, and trajectory-driven volumetric reconstruction. By structurally linking motion measurement with image acquisition and spatial modeling, this framework enables deterministic slice placement and consistent volume formation while minimizing reliance on external tracking or complex calibration procedures. Consequently, the proposed design emphasizes reproducibility, system portability, and reduced calibration complexity, supporting practical deployment in point-of-care breast imaging scenarios.

### 3.1. Dual-Rotary-Encoder Probe Pose Sensing

Accurate trajectory estimation is essential for freehand 3D ultrasound reconstruction. Traditional approaches employ optical tracking, electromagnetic sensors, robotic guidance, or inertial measurement units, all of which introduce cost, environmental sensitivity, or drift. In contrast, the proposed system directly measures probe motion using a mechanically constrained sensing architecture.

Two AS5600 magnetic rotary encoders (Atlas Scientific, Long Island City, NY, USA) were integrated into a custom 3D-printed probe attachment to capture complementary motion components:

- Displacement rotary encoder—coupled to a roller mechanism, converting probe translation into rotational measurements to estimate cumulative travel distance.
- Inclination rotary encoder—mounted on a rocker-type hinge, measuring probe tilt relative to a perpendicular reference axis.

Together, these sensors provide a continuous 2DoF representation of probe motion along the breast surface. This configuration reflects the observation that breast scanning typically occurs along a locally planar manifold with limited rotational variability, making displacement and tilt the primary determinants of spatial frame placement.

**Figure 1:**
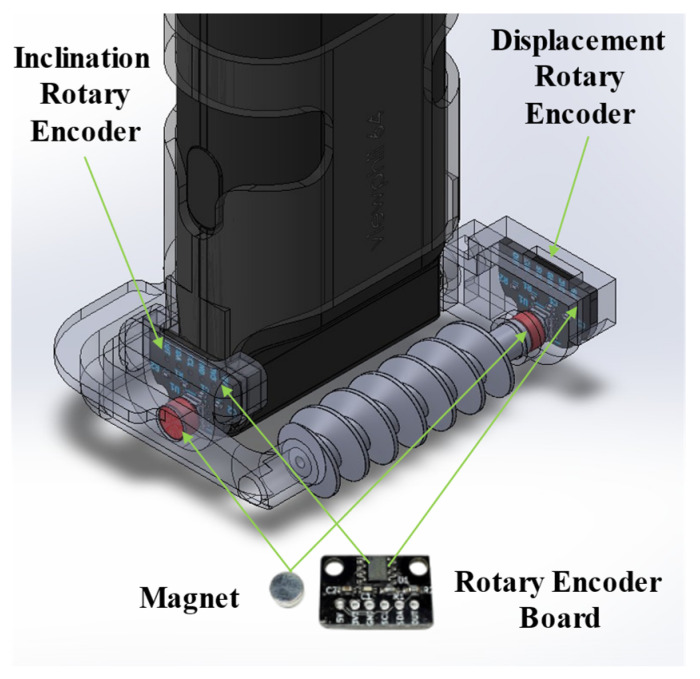
Mechanical configuration of the proposed dual-rotary-encoder sensing module showing displacement and inclination encoder placement.

By measuring motion directly rather than estimating it through inertial integration, the dual-rotary-encoder architecture reduces drift accumulation and improves measurement stability. The sensing module therefore constitutes the central methodological contribution of this work. Figure 1 shows locations of the two rotary encoders used in this study.

### 3.2. Hardware Integration and Synchronized Acquisition

The sensing module was mounted on a commercial ultrasound probe [23] using a rigid 3D-printed frame designed to preserve geometric alignment between the probe, the displacement sensing mechanism, and the inclination hinge. To ensure reliable translation measurement under clinical scanning conditions—where ultrasound gel and soft tissue surfaces can introduce slippage—a slip-resistant roller mechanism was specifically designed. The roller employs a high-friction contact surface and controlled mechanical preload to maintain continuous traction between the probe attachment and the scanning surface. This configuration minimizes motion loss caused by micro-slip, enabling the rotary encoders to capture probe displacement with high fidelity even in gel-coated environments.

**Figure 2:**
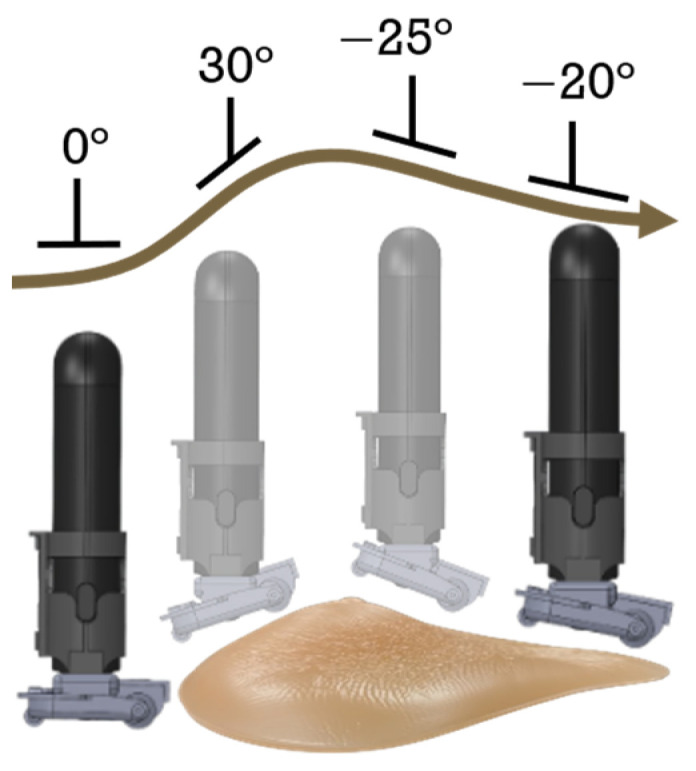
Inclination sensing mechanism showing probe tilt measurement relative to the surface-normal reference axis using the hinge-mounted rotary encoder.

Both rotary encoders were interfaced with a Seed Studio XIAO ESP32S3 [24] microcontroller responsible for angle decoding, incremental displacement computation, timestamp synchronization, and data transmission to a host workstation via USB serial communication. During scanning, ultrasound frames were recorded concurrently with cumulative travel distance, inclination angle, and temporal metadata, allowing each image slice to be associated with a physically measured pose state. Figure 2 illustrates the combined operation of these mechanisms in capturing pose data.

The integration of slip-resistant displacement sensing with synchronized acquisition reduces trajectory uncertainty and improves spatial consistency during volumetric reconstruction. To ensure interoperability with visualization platforms, ultrasound images and pose logs were standardized using the Nearly Raw Raster Data (NRRD) format, preserving geometric spacing and temporal correspondence.

### 3.3. Trajectory Modeling Using Time-Aware Reduced Pose Representation

The probe trajectory was reconstructed sequentially using incremental displacement, inclination, and elapsed time measurements obtained from the dual-rotary-encoder sensing system. Rather than assuming constant probe motion, the proposed framework incorporates temporal information to account for variations in scanning speed and frame acquisition intervals, thereby improving spatial consistency during reconstruction.

Let $t_{n}$ denote the elapsed time associated with the *n*-th ultrasound frame with *n* >= 0, $r_{n}$ the incremental travel distance measured by the displacement rotary encoder between consecutive timestamps ${(t}_{n-1},t_{n})$, and $\theta_{n}$ the inclination angle measured by the angular rotary encoder at time $t_{n}$ in radians. The inclination angle $\theta_{n}$ is defined relative to the initial inclination measured at $t_{0}$, such that $\theta_{n}$ represents the angular deviation from the starting orientation of the probe. The probe position is updated iteratively according to a time-indexed polar-to-Cartesian transformation:

(1) $$x_{n}=x_{n-1}{+r}_{n}cos(\theta_{n})$$

(2) $$y_{n}=y_{n-1}{+r}_{n}sin(\theta_{n})$$

where the incremental displacement $r_{n}$ is derived from rotary encoder rotation accumulated over the time interval ${∆t_{n}=t}_{n}-t_{n-1}$. This formulation enables the trajectory to reflect actual probe motion rather than an assumed constant sampling rate.

Incorporating elapsed time provides two advantages. First, it allows synchronization between ultrasound frame acquisition and pose updates, ensuring that each image slice is placed at the correct spatial location even under irregular scanning speeds. Second, it enables detection of anomalous motion events, such as rapid probe movement or temporary contact loss, which manifest as large displacement changes over short time intervals. These events can be identified and handled during reconstruction to reduce spatial artifacts. To characterize scanning dynamics, instantaneous probe velocity was computed as:

(3) $$v_{n}=\frac{r_{n}}{{∆t}_{n}}$$

where $v_{n}$ represents the translational speed between consecutive frames. Velocity estimation serves two purposes. First, it provides an additional descriptor of operator behavior during acquisition. Second, it enables identification of motion irregularities that may degrade volumetric reconstruction. Because breast scanning is typically performed at relatively low and smooth speeds, abrupt velocity changes can indicate frame skipping, temporary loss of surface contact, or mechanical slip.

Trajectory reliability depends on maintaining consistent motion. Therefore, a time-aware outlier detection strategy was implemented based on displacement and velocity statistics. An observation was considered anomalous when either:

- The incremental displacement $r_{n}$ exceeded twice the median displacement observed within a local temporal window, or
- The estimated velocity $v_{n}$ exceeded a predefined physiological scanning threshold.

Such anomalies typically correspond to rapid probe motion, rotary encoder discontinuities, or micro-slip events at the probe–surface interface. Detected outliers were flagged during preprocessing and excluded or interpolated prior to volumetric reconstruction to reduce spatial artifacts.

The combination of time-aware trajectory modeling, velocity estimation, and anomaly handling produces a robust representation of probe motion that remains consistent under realistic scanning conditions. Importantly, these procedures operate within the reduced 2DoF pose framework, demonstrating that reliable spatial reconstruction can be achieved using a reduced-dimensional pose representation under constrained breast scanning conditions.

### 3.4. Trajectory-Driven Volumetric Reconstruction

**Figure 3:**
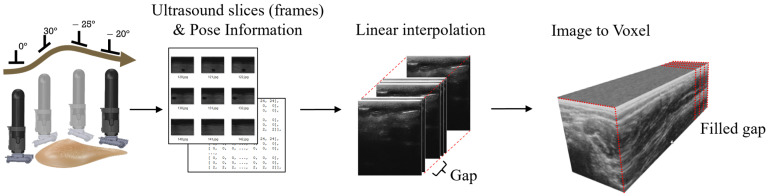
Trajectory-driven volumetric reconstruction workflow integrating pose estimation, ultrasound slice placement, spatial interpolation, and voxel generation within 3D Slicer. The red line illustrates the filled gap area.

Volumetric reconstruction was performed by spatially embedding ultrasound frames along the probe trajectory estimated from the dual-encoder sensing framework. As illustrated in Figure 3, the reconstruction process follows a sequential pipeline that integrates pose estimation, frame placement, and voxel resampling to produce a continuous three-dimensional representation.

Each ultrasound slice was associated with a time-indexed pose state derived from displacement and inclination measurements. Using the reconstructed trajectory described in Section 3.3, frames were positioned in spatial coordinates such that their relative placement reflected the actual probe motion across the breast surface. This trajectory-driven positioning transforms the originally independent two-dimensional ultrasound slices into an irregularly sampled volumetric dataset.

As depicted in Figure 3, the resulting slice distribution does not initially form a uniform grid due to variations in scanning speed and trajectory curvature. Therefore, a resampling stage was applied in which voxel intensities were estimated through linear interpolation between neighboring slices. This interpolation fills spatial gaps while preserving structural continuity, enabling generation of a geometrically consistent volume.

The reconstruction workflow was implemented in 3D Slicer V5.2.2 [25], where pose-derived spacing parameters were incorporated into volumetric nodes to maintain accurate scaling and orientation. The rotary encoder-driven trajectory ensures stable frame placement, reducing slice misalignment commonly observed in IMU-based freehand reconstruction. Consequently, the pipeline shown in Figure 3 demonstrates how reduced-dimension pose sensing can be translated into reliable volumetric imaging without external tracking infrastructure.

By explicitly linking trajectory estimation to slice placement and interpolation, the reconstruction framework provides a deterministic pathway from raw ultrasound acquisition to three-dimensional visualization, supporting reproducible analysis of internal breast structures.

### 3.5. Rationale for Reduced 2DoF Pose Modeling in Breast Scanning

The sensing framework is designed based on the observation that breast ultrasound scanning occurs under constrained motion conditions in which probe movement is largely limited to surface-following translation with controlled inclination adjustments. Continuous probe–tissue contact restricts out-of-plane motion and stabilizes orientation, allowing the scanning process to be approximated as motion along a locally smooth surface. Under these conditions, displacement along the scanning path and tilt relative to the surface normal constitute the primary determinants of spatial slice placement.

Accordingly, the proposed system adopts a reduced pose representation consisting of translational displacement and inclination. By aligning sensing dimensionality with the intrinsic motion characteristics of the scanning task, the framework simplifies pose estimation while retaining the spatial information required for volumetric reconstruction. Rotational components such as yaw and roll are not explicitly modeled in the present framework because their influence on slice positioning is relatively limited under routine surface-constrained breast scanning conditions.

Within this methodological design, trajectory estimation is expressed as a time-indexed sequence of displacement–inclination pairs that defines probe motion across the scanned surface. This representation provides the foundation for slice placement, interpolation, and volume formation described in Section 3.4. The framework models probe motion using displacement and inclination measurements, enabling reproducible volumetric imaging while avoiding the complexity associated with full six-degree-of-freedom tracking systems.

The proposed framework is specifically designed for surface-constrained breast ultrasound scanning, where probe motion is largely restricted to translational movement along the tissue surface with limited angular variation. Therefore, the reduced 2DoF representation should not be interpreted as a universal replacement for full 6DoF tracking in unconstrained freehand ultrasound applications. More complex anatomical regions or acquisition scenarios involving substantial probe rotation and out-of-plane motion may still require higher-dimensional tracking approaches.

## 4. Evaluation and Results

The proposed portable 3D ultrasound system was evaluated to verify the reliability of the dual-rotary-encoder pose sensing framework and the accuracy of the trajectory-driven volumetric reconstruction pipeline. Two aspects were investigated: (i) probe pose estimation accuracy and stability and (ii) reconstruction accuracy using a breast phantom with known geometric properties. Pose estimation performance was assessed through repeated measurements conducted under controlled conditions to minimize external variability. Measured displacement and inclination were compared against ground-truth values, and quantitative error metrics were computed to characterize precision, repeatability, and temporal stability. Reconstruction accuracy was evaluated using a breast phantom designed to replicate breast tissue structure. The BB-03 phantom (OST Corporation) [26], which contains spherical inclusions of known diameters, served as the reference object, and reconstructed volumes were quantitatively compared with these known dimensions to assess geometric fidelity and visualization accuracy. This evaluation framework provides a controlled yet clinically relevant validation of the proposed system, supporting its potential application in portable breast imaging where reliable probe tracking and accurate volumetric visualization are essential for lesion assessment and longitudinal monitoring.

### 4.1. Experimental Design

The evaluation comprised two experiment groups: pose information measurement and visualization assessment. Pose measurement experiments included positional displacement and angular displacement tests designed to evaluate the system’s capability to capture probe translation and orientation. Positional displacement was examined under two contact conditions: fabric-only and fabric with ultrasound gel, to evaluate motion fidelity and the effectiveness of the slip-resistant roller mechanism. Angular displacement experiments used controlled tilt motion generated by a robotic arm, enabling comparison between the rotary encoder-based sensing approach and a conventional IMU sensor using consistent ground-truth measurements.

The visualization experiment involved scanning a breast phantom containing spherical inclusions of known size. Multiple acquisition trials were performed, and reconstructed volumes were analyzed to determine inclusion size and spatial consistency. Deviations between actual and reconstructed measurements were used as performance indicators to evaluate the influence of trajectory estimation on volumetric reconstruction accuracy.

### 4.2. Pose Measurement Experiments

#### 4.2.1. Positional Displacement

**Figure 4:**
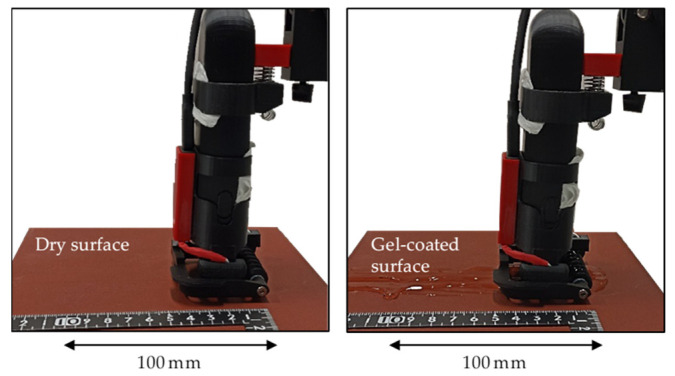
Experimental setup for the positional displacement test using a robotic arm to generate controlled translational motion.

The positional displacement experiment evaluated the distance measurement technique implemented through the slip-resistant roller mechanism. Tests were conducted on two 100 mm flat surfaces: a leather surface representing dry acquisition and a gel-coated surface representing realistic scanning conditions (Figure 4). To minimize variability, a Dobot Magician robotic arm was used to translate the device along predefined trajectories. This setup enabled repeatable measurement of positional shifts and allowed assessment of the roller mechanism’s ability to maintain motion fidelity under gel contact.

**Figure 5:**
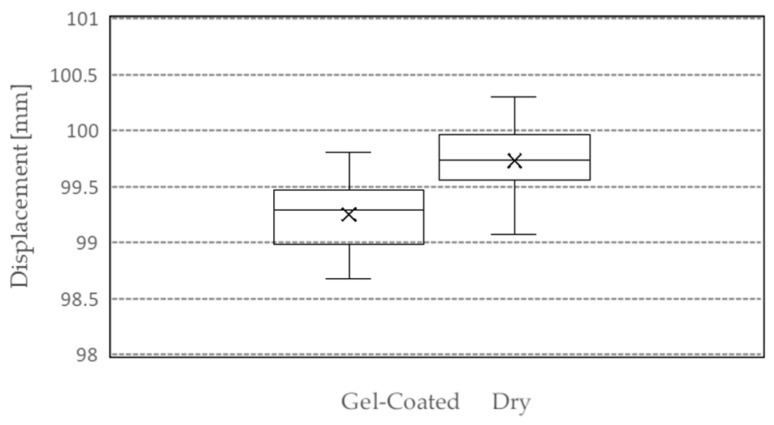
The box-and-whisker plot of positional displacement measurements performed on the dry and gel-coated surface (*n* = 30). Cross markers (×) indicate mean values.

The true reference displacement was set to 100 mm. Thirty repeated trials were performed for each surface condition. Figure 5 shows the box-and-whisker plot of both measurement results. Measurements on the dry surface yielded a root mean square error (RMSE) of 0.38 mm, whereas measurements on the gel-coated surface resulted in an RMSE of 0.81 mm. The measurements obtained under the Gel-coated and dry surfaces were compared using a paired-samples t-test (*n* = 30). The mean under the gel-coated condition (99.25 mm) was lower than that under the dry condition (99.73 mm), despite similar within-condition variances (0.091 mm vs. 0.074 mm). Under the null hypothesis of no mean difference, the test yielded *t*(29) = −6.21 with a two-tailed *p*-value of 8.96 × 10^−7^, indicating a statistically significant difference between conditions.

Although both conditions exhibited relatively small slip errors with respect to the 100 mm reference displacement, the larger deviation observed under the gel-coated condition demonstrates that the presence of ultrasound gel induces significantly greater slip error than the dry surface. Nevertheless, the results confirm millimeter-level positional accuracy and indicate that the slip-resistant roller mechanism maintains reliable displacement sensing even under gel-mediated contact conditions. Importantly, this level of translational precision directly contributes to improved slice placement during volumetric reconstruction, thereby reducing cumulative trajectory error and supporting geometrically consistent three-dimensional visualization.

#### 4.2.2. Angular Displacement

**Figure 6:**
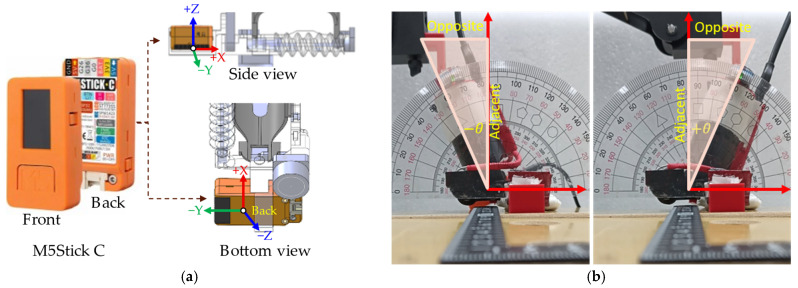
Experimental setup for the angular displacement experiment using the Dobot robotic arm to generate controlled inclination motion (±*θ*). (**a**) Alignment of the proposed rotary encoder-based system and the reference IMU sensor (M5Stick C) along the same central axis. The arrow shows how the IMU is viewed from different angles. (**b**) Right-triangle model for converting the robotic arm’s linear displacement into inclination angle.

The angular displacement experiment evaluated the accuracy and stability of inclination sensing. Controlled tilt motion was generated using the Dobot robotic arm to ensure repeatability and to provide ground-truth reference data. The proposed rotary encoder-based system and a reference IMU sensor (M5Stick C) [27] were aligned along the same central axis, enabling direct comparison under identical motion conditions (Figure 6a). Because the robotic arm produces linear displacement rather than direct angular output, a geometric conversion based on a right-triangle model was employed to derive inclination values (Figure 6b). The ground-truth angle was computed as

(4) $$\theta =\tan^{-1}\left(\frac{Opposite}{Adjacent}\right)$$

**Figure 7:**
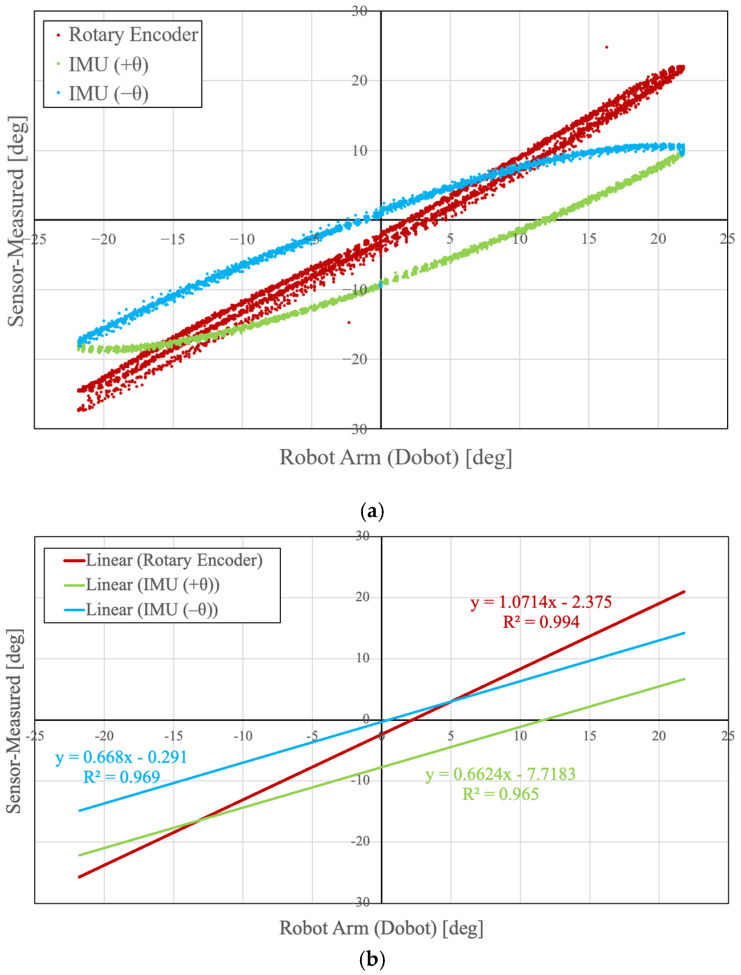
Scatter plots and linear regression analysis of sensor measurements versus Dobot angle. The rotary encoder shows high linearity and accuracy, while the IMU exhibits direction-dependent bias and reduced sensitivity. (**a**) Scatter plots comparing measured inclination values against Dobot ground-truth angles. (**b**) Scatter plots comparing measured inclination values against Dobot ground-truth angles.

Thirty repeated trials were conducted to evaluate measurement accuracy. Inclination measurements were obtained by actuating the mechanism to swing both devices over a controlled angular range of approximately −22° to +22°. Figure 7 shows the scatter plots and linear regression results comparing the sensor measurements with the Dobot angle.

The rotary encoder demonstrates a strong linear relationship with the reference angle, with a regression equation of y = 1.071x − 2.375 and a high coefficient of determination (R^2^ = 0.994). This result indicates excellent agreement and high measurement accuracy across the entire range of motion. In contrast, the IMU measurements show lower linearity and noticeable deviations depending on the direction of motion. For the positive direction (+θ), the regression is given by *y* = 0.662*x* − 7.718 with *R*^2^ = 0.965, while for the negative direction (−θ), the regression is *y* = 0.668*x* − 0.291 with *R*^2^ = 0.969. In both cases, the slopes are significantly lower than the ideal value of 1, indicating systematic underestimation of the angle. Furthermore, the IMU (+θ) exhibits a larger offset error, as reflected by its substantial negative intercept, suggesting the presence of bias or calibration drift. Although the IMU maintains moderate linearity (R^2^ ≈ 0.97), its performance is inferior to that of the rotary encoder in terms of both accuracy and consistency.

The rotary encoder achieved an RMSE of 2.76°, whereas the IMU sensor exhibited higher RMSE values of 4.57° and 8.95°, further confirming the superior performance of the rotary encoder. This improvement reflects the advantage of direct mechanical sensing, which avoids the cumulative integration errors and drift commonly observed in inertial measurement units. Improved inclination stability directly contributes to more accurate slice alignment during freehand acquisition, thereby reducing orientation-induced artifacts and enhancing volumetric reconstruction consistency.

**Figure 8:**
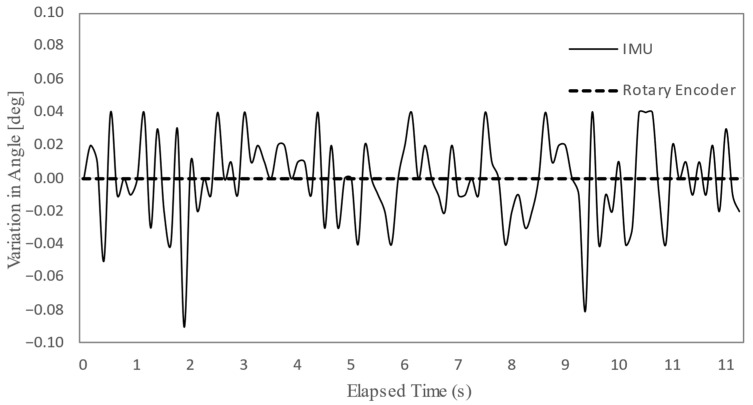
Time-series comparison of inclination measurements obtained from the rotary encoder-based system and the IMU sensor under stationary conditions, demonstrating the improved stability and reduced jitter of the proposed system.

In addition to accuracy, measurement stability was evaluated under stationary conditions by continuously recording inclination data. As illustrated in Figure 8, the proposed system maintained stable measurements with minimal fluctuation, whereas the IMU sensor exhibited noticeable jitter. These results demonstrate that rotary encoder-based inclination sensing provides both higher accuracy and greater temporal stability, which are critical for reducing orientation-induced slice misalignment in freehand ultrasound reconstruction.

### 4.3. Phantom-Based Volumetric Reconstruction Evaluation

The phantom-based volumetric reconstruction experiment was conducted to evaluate the geometric fidelity and reconstruction consistency of the proposed trajectory-driven freehand 3D ultrasound framework. In contrast to the pose measurement experiments presented in Section 4.2.1 and Section 4.2.2, this evaluation focused on the cumulative influence of probe trajectory estimation, slice placement, interpolation, and segmentation on the final reconstructed volumetric representation.

**Figure 9:**
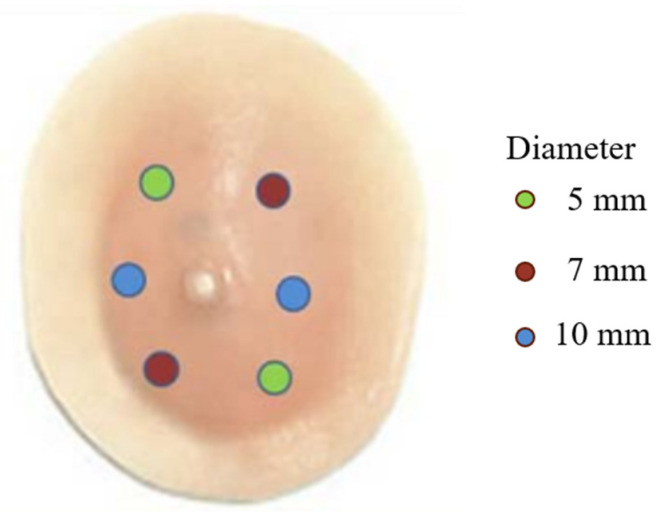
Illustration of breast phantom with spherical inclusions of predefined diameters used as ground-truth targets for volumetric reconstruction evaluation. The inclusions consist of spheres with diameters of 5 mm, 7 mm, and 10 mm.

A breast phantom (BB-03, OST Corporation, Japan) containing six spherical inclusions with predefined diameters was used as the reference object (Figure 9). The phantom included two inclusions, each with diameters of 5 mm, 7 mm, and 10 mm, positioned at depths ranging from 10 mm to 20 mm. These inclusions provided repeated reference targets for evaluating reconstruction accuracy and reproducibility across multiple object scales.

**Figure 10:**
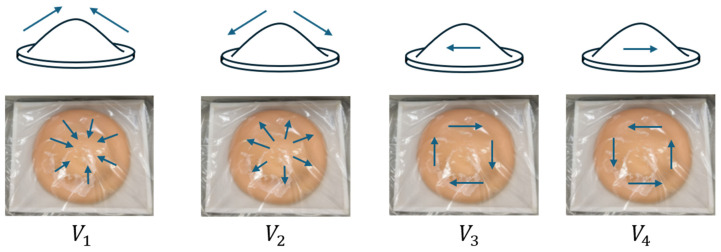
Scanning trajectory patterns used for volumetric acquisition: radial inward (*V*_1_), radial outward (*V*_2_), clockwise circumferential (*V*_3_), and counterclockwise circumferential (*V*_4_), designed to evaluate directional effects on reconstruction accuracy. The arrow in the image indicates the direction of the scan.

Data acquisition was performed using four scanning trajectories, denoted as $V_{1}\sim V_{4}$, representing radial inward, radial outward, clockwise circumferential, and counterclockwise circumferential scanning patterns, respectively (Figure 10). For each inclusion size and scanning trajectory, volumetric acquisition and reconstruction were repeated ten times, resulting in a total of 120 reconstructed samples. These trajectories were designed to evaluate whether scanning trajectories influence volumetric reconstruction accuracy while maintaining comparable spatial sampling density. The phantom was positioned to simulate a supine patient configuration, and scanning was performed by a seated operator using a consistent posture to reduce operator-induced variability.

Following acquisition, volumetric reconstruction was performed in 3D Slicer using the trajectory-driven slice placement framework described in Section 3.4. Ultrasound slices were spatially embedded according to the estimated probe trajectory and resampled into a three-dimensional voxel space with a voxel spacing of 0.1 mm.

Segmentation of the reconstructed inclusions was performed using a semi-automatic thresholding approach in 3D Slicer. A fixed thresholding range of 0–50 was applied uniformly across all reconstructed volumes. Any subsequent manual intervention was limited to segmentation refinement and did not involve adjustment of the threshold values. A formal sensitivity analysis of the reconstructed volumes with respect to threshold selection was not performed.

After segmentation, the diameter and volume of each reconstructed inclusion were estimated using the Segment Statistics module in 3D Slicer. Theoretical sphere volumes were calculated from the known inclusion diameters (*r*) using

(5) $$V=\frac{4}{3}\pi r^{3}.$$

Reconstruction accuracy was evaluated using the absolute error (AE)

(6) $$\mathrm{AE}=\left|V_{\mathrm{reconstructed}}-V_{\mathrm{theoretical}}|\right.$$

and the relative error (RE)

(7) $$RE=\frac{\left|V_{reconstructed}-V_{theoretical}\right|}{V_{theoretical}}\times 100\%.$$

where $V_{reconstructed}$ and $V_{theoritical}$ denote the reconstructed and theoretical inclusion volumes, respectively. These measurements reflect the cumulative influence of trajectory estimation, slice placement, interpolation, and segmentation accuracy on volumetric reconstruction performance.

**Figure 11:**
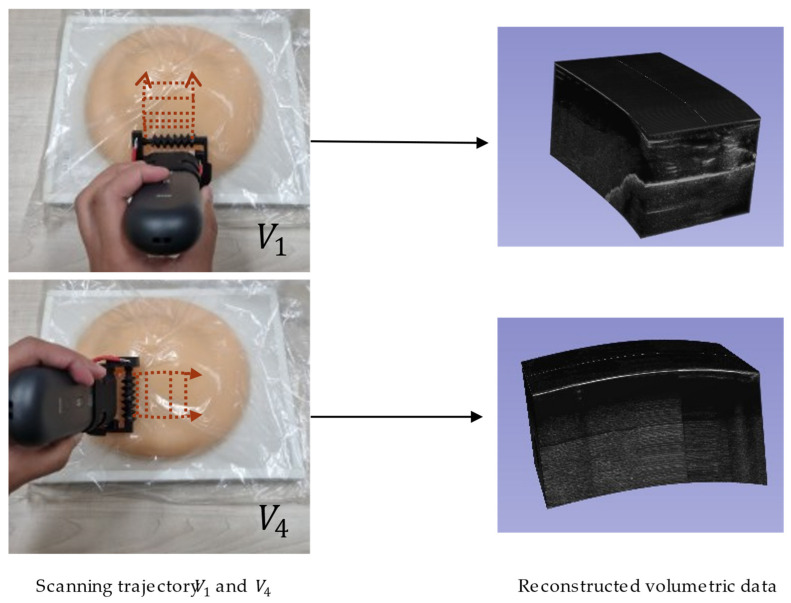
Visualization of the *V*_1_ and *V*_4_ acquisition trajectory alongside the reconstructed volumetric data, illustrating the relationship between probe motion and 3D reconstruction. The dashed arrow indicates the scanning direction.

For each inclusion size and scanning trajectory, data acquisition and reconstruction were repeated ten times, resulting in a total of 120 reconstructed samples. Figure 11 illustrates the $V_{1}$ and $V_{4}$ scanning trajectory together with the corresponding reconstructed volumetric data. Quantitative evaluation focused on comparing reconstructed volume consistency across object sizes and scanning methods. Because segmentation was performed by a single operator under consistent threshold selection criteria, inter-observer variability was not evaluated in the present study. Future work will investigate multi-observer segmentation consistency and automated threshold optimization to improve reproducibility under clinical conditions.

In addition to descriptive statistics, a two-way analysis of variance (ANOVA) was conducted to investigate the effects of inclusion diameter and scanning trajectory on reconstruction error. Statistical significance was defined as *p* < 0.05.

#### 4.3.1. Outlier Detection Statistics

**Figure 12:**
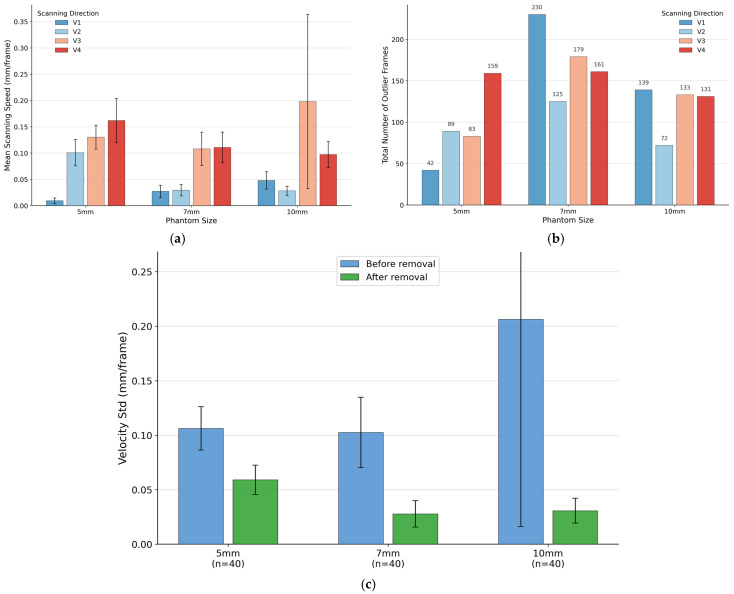
Scanning velocity statistics and velocity outlier detection results. These statistics demonstrate that the proposed outlier detection method effectively improved the stability and consistency of the scanning trajectory. (**a**) Mean Scanning Speed per Size, (**b**) Total Outlier Count, (**c**) Velocity Std: Before vs. After Outlier Removal.

After data acquisition, the scanning velocity data were analyzed together with the performance of the proposed velocity-based outlier detection method to evaluate its effectiveness in improving trajectory stability. Figure 12a presents the scanning speed statistics for all experimental conditions. The results show that the average scanning speed varied depending on both the inclusion size and the scanning method. During the reconstruction process, the number of detected velocity outliers was quantified, as shown in Figure 12b. A considerable number of outliers were observed across all datasets, indicating that irregular probe motion occurred consistently during manual scanning.

After applying the proposed velocity-based outlier detection and correction method, a substantial reduction in the standard deviation of the scanning velocity was observed, particularly for the 10 mm inclusion experiments, as shown in Figure 12c. This result indicates that the proposed outlier detection method effectively improved the stability and consistency of the scanning trajectory, providing a more reliable input for the subsequent reconstruction process.

#### 4.3.2. Quantitative Reconstruction Accuracy

Quantitative evaluation focused on estimating the volume of each reconstructed inclusion to assess reconstruction accuracy across object scales and acquisition patterns. For each sample, the theoretical volume was calculated from the known diameter, and the relative difference between the measured and theoretical volumes was computed as the reconstruction error. Table 2 summarizes the reconstruction results obtained for each inclusion diameter and scanning trajectory. Mean reconstruction error and standard deviation were calculated from ten repeated trials for each condition.

A two-way ANOVA was performed to evaluate the effects of inclusion diameter and scanning trajectory on the relative reconstruction error. The analysis included three inclusion diameters (5 mm, 7 mm, and 10 mm) and four scanning trajectories ($V_{1}\sim V_{4}$), with ten repeated measurements for each experimental condition. The dependent variable was the relative reconstruction error, while the independent variables consisted of inclusion diameter and scanning trajectory. The analysis examined both the main effects of each factor and their interaction effect to determine whether reconstruction performance depended on object size, acquisition trajectory, or their combination.

The normality assumption was assessed by applying the Shapiro–Wilk test to the residuals of the ANOVA model, and homogeneity of variances was evaluated using Levene’s test. When significant main effects or interaction effects were identified, post hoc multiple comparisons were performed using Tukey’s honestly significant difference (HSD) test. Statistical significance was defined as *p* < 0.05.

The results of the assumption tests are summarized in Table 3. Prior to conducting the two-way ANOVA, the underlying assumptions were evaluated. The Shapiro–Wilk test performed on the ANOVA residuals indicated that the residuals were normally distributed (W = 0.992, *p* = 0.732), satisfying the normality assumption. Furthermore, Levene’s test showed no significant difference in group variances (F = 1.761, *p* = 0.069), indicating that the assumption of homogeneity of variances was also satisfied. These results confirmed that the data met the assumptions required for applying a parametric two-way ANOVA.

The ANOVA results are summarized in Table 4. The ANOVA revealed that object size had a statistically significant effect on reconstruction accuracy (F(2, 108) = 3.30, *p* = 0.041). This result indicates that reconstruction performance differed significantly across object sizes. Scanning trajectories also showed a statistically significant main effect on reconstruction accuracy (F(3, 108) = 8.37, *p* < 0.001), suggesting that reconstruction performance varied depending on the scanning trajectories used. In addition, a statistically significant interaction effect was observed between object size and scanning trajectories (F(6, 108) = 2.75, *p* = 0.016). This interaction indicates that the effect of scanning trajectories on reconstruction accuracy depends on the size of the object being reconstructed.

Post hoc analysis was conducted using Tukey’s Honestly Significant Difference (HSD) test to determine which scanning trajectories differed significantly in relative reconstruction error within each inclusion diameter after the two-way ANOVA. Because the diameter × trajectory interaction was significant, the post hoc results were interpreted as simple effects rather than as overall trajectory differences pooled across all diameters. The Tukey HSD comparisons are summarized in Table 5. For the 5 mm inclusions, V4 exhibited significantly higher relative error than both V1 and V3 (*p* < 0.05), whereas the remaining pairwise differences were not significant. For the 7 mm inclusions, V1 showed significantly lower relative error than V2 and V4 (*p* < 0.05). In contrast, for the 10 mm inclusions, no statistically significant pairwise differences were observed among the four trajectories. Overall, these findings indicate that the effect of scanning trajectory on relative reconstruction error depends on inclusion diameter, supporting the presence of a meaningful interaction between the two factors.

Overall, the results indicate that relative reconstruction error is significantly influenced by both object size and scanning trajectory. Furthermore, the significant interaction between these factors indicates that the effect of scanning trajectory varies with object size, suggesting that the optimal acquisition strategy may depend on the size of the target to achieve improved volumetric reconstruction accuracy in freehand ultrasound imaging.

#### 4.3.3. Visual Assessment of Reconstructed Volumes

Volumetric reconstruction performed using 3D Slicer demonstrated that the proposed device was able to clearly visualize the spherical inclusions within the breast phantom. The reconstructed spherical morphology varied depending on object size. Larger inclusions, particularly those with a diameter of 10 mm, appeared more distinct due to increased voxel representation and improved spatial sampling. In contrast, smaller inclusions, such as those measuring 5 mm, were more difficult to visualize clearly, reflecting the higher sensitivity of small structures to interpolation and trajectory sampling density.

**Figure 13:**
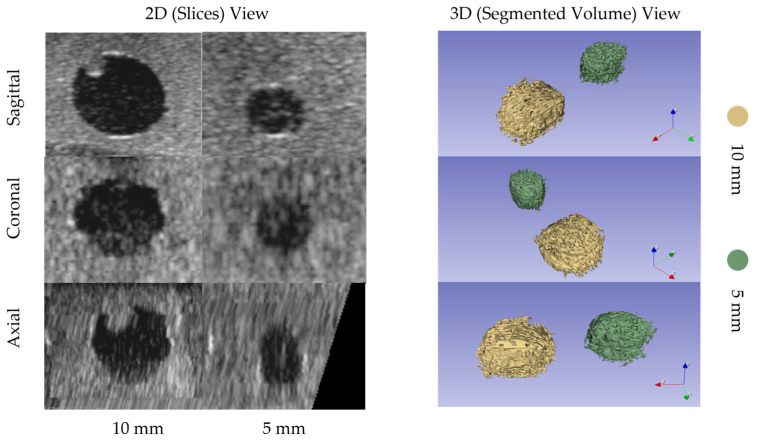
Representative volumetric reconstruction results for spherical inclusions of different sizes (5 mm and 10 mm) generated using the proposed system.

Representative visualization results for the 5 mm and 10 mm inclusions are shown in Figure 13. Despite differences in mean reconstruction error and variability across object sizes, the developed device and reconstruction pipeline consistently produced volumetric representations that closely resembled the true shape and spatial distribution of the inclusions. These qualitative observations complement the quantitative analysis, indicating that the rotary encoder-based trajectory estimation provides sufficient spatial information to support visually coherent volumetric reconstruction across varying object scales.

#### 4.3.4. Pose Measurement Against Independent Reference

**Figure 14:**
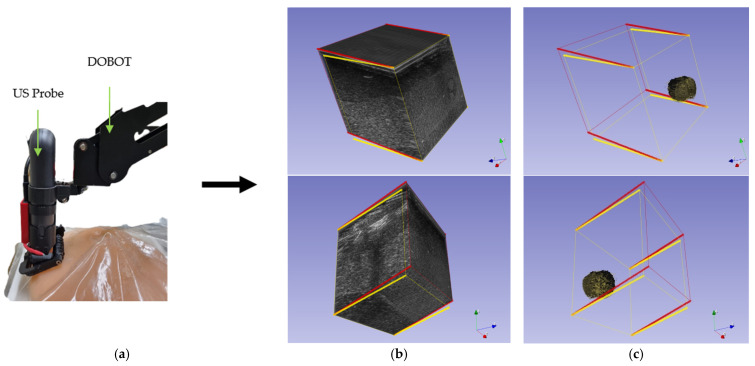
Comparison between the reconstructed volumetric results obtained using the proposed encoder-based freehand ultrasound framework and the Dobot ground-truth reference. The reconstructed 3D ultrasound volume and segmented spherical inclusion are visualized together. The red color indicates the Dobot reference geometry, while the yellow contour represents the proposed reconstruction result. (**a**) Probe Placement with DOBOT, (**b**) The reconstructed volumetric results, (**c**) The segmented spherical inclusions.

To provide qualitative evaluation of the reconstructed three-dimensional geometry, additional volumetric data were acquired together with the corresponding probe trajectories. Using one of the acquisition methods ($V_{1}$), probe motion was controlled using a DOBOT robotic arm, enabling generation of a ground-truth trajectory from the predefined robotic motion path. The resulting volumetric reconstruction and segmentation results are shown in Figure 14. Figure 14b presents the reconstructed ultrasound data visualized as a complete three-dimensional volume. Figure 14c further illustrates the segmented spherical inclusion extracted from the reconstructed volume together with contour-based comparison against the ground-truth reference. In the visualization, the red contour represents the reference geometry reconstructed from the DOBOT trajectory, whereas the yellow contour represents the reconstruction obtained using the proposed encoder-based freehand ultrasound framework.

**Figure 15:**
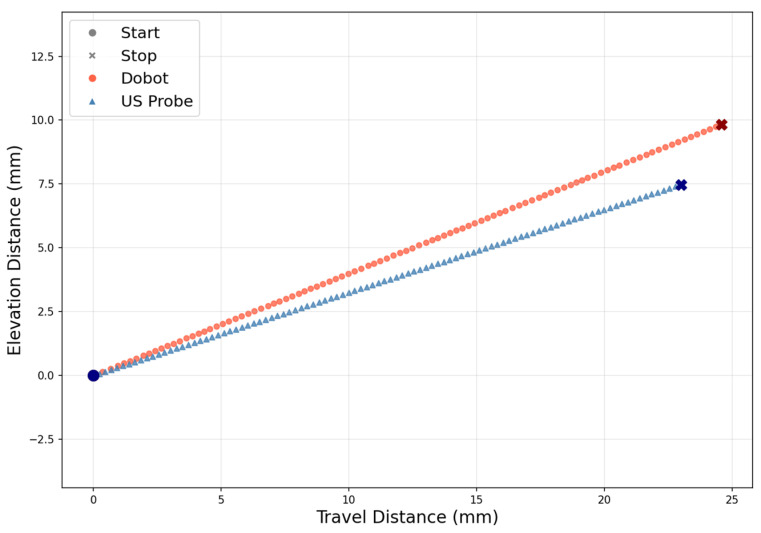
Trajectory comparison between DOBOT vs. Ultrasound Probe.

As shown in Figure 15, the comparison demonstrates that the proposed system reconstructs the spherical inclusion with geometry and spatial distribution closely matching the ground-truth reference. The reconstructed object preserves an approximately spherical morphology with only minor boundary deviation, indicating good volumetric reconstruction fidelity and spatial consistency.

The trajectory comparison was performed under robot-driven motion using the V1 scanning trajectory to evaluate the proposed sensing system against an independent reference. While the reconstructed trajectory closely followed the overall motion pattern of the Dobot, differences in displacement magnitude were observed, particularly along the vertical axis (9.83 mm for the Dobot and 7.48 mm for the proposed system). This difference is expected because the two systems measure different physical quantities. The Dobot reports the position of the ultrasound probe, whereas the proposed sensing system estimates the travel distance of the encoder wheel in contact with the phantom surface. Consequently, this experiment evaluates the consistency of the reconstructed trajectory rather than absolute positional agreement between the two measurement systems.

#### 4.3.5. Underlying Cause of Size Significance

During volumetric reconstruction, each ultrasound frame was positioned in three-dimensional space using pose estimation derived from displacement and inclination measurements. Reconstruction quality was influenced by both motion-related factors and image-based uncertainties.

Although motion-induced outliers may introduce localized geometric distortions and surface discontinuities, the statistical analysis indicates that inclusion size also significantly affects the relative volumetric error. Larger inclusions generally exhibited lower relative errors than smaller inclusions. This trend is expected because a comparable absolute reconstruction deviation corresponds to a smaller percentage of the true volume as object size increases.

In addition, smaller inclusions are inherently more sensitive to ultrasound spatial resolution, interpolation uncertainty during volumetric reconstruction, and segmentation inaccuracies. These factors amplify the relative error even when the absolute reconstruction deviation remains relatively small.

The significant effect of scanning trajectory further indicates that reconstruction accuracy depends not only on inclusion size but also on the scanning trajectory. Therefore, the observed reconstruction variability is likely the combined result of object size, scanning trajectory, and motion-induced interpolation errors rather than a single dominant factor.

These findings emphasize the importance of maintaining consistent scanning speed and incorporating outlier detection and trajectory smoothing strategies to improve reconstruction stability in portable freehand ultrasound systems.

## 5. Discussion

The results of this study demonstrate that mechanically constrained reduced-dimensional pose sensing can provide stable and sufficiently accurate trajectory information for freehand 3D breast ultrasound reconstruction. The proposed dual-rotary-encoder framework achieved consistent positional and angular measurements while maintaining a compact and portable system configuration. Rather than relying on full six-degree-of-freedom tracking, the framework focuses on the dominant motion characteristics of breast scanning, namely translational movement along the skin surface and limited probe inclination.

The positional displacement experiments showed that the slip-resistant roller mechanism maintained high translational accuracy under both dry and gel-coated conditions. Although the gel-coated surface produced a statistically significant increase in measurement error, the overall deviation remained relatively small. This suggests that direct mechanical displacement sensing can preserve reliable motion estimation even in the presence of ultrasound gel, which is a common source of slippage in practical scanning environments. The results also indicate that translational measurement errors are unlikely to become a dominant source of reconstruction degradation under typical scanning conditions.

The angular displacement experiments further highlighted the advantages of encoder-based sensing compared with IMU-based estimation. The rotary encoder demonstrated higher linearity, lower RMSE, and improved temporal stability. In contrast, the IMU exhibited direction-dependent bias and noticeable measurement jitter. These findings are consistent with the known susceptibility of inertial sensors to drift accumulation and bias instability. Because freehand ultrasound reconstruction depends strongly on stable slice orientation, improved inclination sensing likely contributed to the consistent volumetric reconstruction observed in the phantom experiments.

The phantom reconstruction results demonstrated that the proposed framework was capable of reconstructing spherical inclusions across multiple acquisition trajectories with relatively consistent geometric representation. Statistical analysis showed that reconstruction accuracy was significantly influenced by both inclusion size and scanning trajectory, with a significant interaction between these factors, indicating that the effect of scanning trajectory depended on the inclusion size. Larger inclusions generally exhibited lower relative errors than smaller inclusions, which is expected because a comparable absolute deviation represents a smaller percentage of the true volume as object size increases. In addition, the larger relative errors observed for smaller inclusions are likely associated with the limited spatial resolution of ultrasound imaging, interpolation uncertainty, and increased sensitivity to segmentation inaccuracies.

An important implication of this study is the potential value of task-specific pose modeling in medical imaging systems. Conventional freehand ultrasound systems frequently adopt general-purpose 6DoF tracking architectures originally designed for unconstrained spatial motion. However, breast ultrasound scanning is inherently constrained by continuous probe–surface contact and relatively smooth scanning trajectories. Under such conditions, simplified motion representations may provide sufficient spatial information while substantially reducing hardware complexity and calibration requirements. The present results support this hypothesis and suggest that reduced-dimensional sensing strategies may be suitable for other constrained freehand imaging applications.

Despite these promising findings, several limitations remain. First, the experiments were performed using a breast phantom under controlled laboratory conditions. Clinical breast tissue exhibits more complex deformation, curvature, and operator-dependent variability, which may affect trajectory estimation and reconstruction accuracy. Second, segmentation of reconstructed inclusions was performed semi-automatically using threshold-based adjustment, which introduces operator dependency and may affect quantitative reproducibility. Third, the proposed 2DoF representation does not explicitly model out-of-plane motion or rotational components such as yaw and roll. Although these components appear to have limited influence under constrained scanning conditions, more complex probe motion may require additional sensing dimensions to maintain reconstruction fidelity.

To bound the contribution of yaw and roll within the 40-mm image width, we performed a simple geometric worst-case analysis. The maximum lateral deviation at the image boundary is e=rsinθ in millimeters. This yields 1.74 mm at 5° and 3.47 mm at 10°, indicating that the effect of small angular deviations remains limited under the constrained scanning protocol used in this study.

Although the proposed framework demonstrates the feasibility of freehand 3D ultrasound reconstruction using a reduced-dimensional mechanical sensing approach, the current volumetric errors indicate that further improvements in trajectory estimation, image processing, and segmentation are required before the system can be considered for quantitative longitudinal lesion monitoring in clinical practice.

Future work should therefore focus on clinical validation under realistic scanning conditions, automated segmentation and reconstruction optimization, and evaluation of inter-observer reproducibility. Integration of adaptive motion correction or hybrid sensing approaches may further improve robustness in situations involving complex tissue geometry or irregular probe motion. In addition, real-time reconstruction feedback could potentially assist operators in maintaining stable scanning trajectories during freehand acquisition.

## 6. Conclusions

This study presented a portable freehand 3D ultrasound framework for breast imaging based on a dual-rotary-encoder two-degree-of-freedom pose sensing mechanism. The proposed system combines translational displacement sensing, inclination measurement, time-aware trajectory modeling, and trajectory-driven volumetric reconstruction within a compact mechanically constrained architecture.

Experimental evaluation demonstrated that the proposed sensing framework achieved millimeter-level positional accuracy under both dry and gel-coated scanning conditions, with RMSE values of 0.38 mm and 0.81 mm, respectively. Inclination sensing using the rotary encoder showed higher accuracy and improved temporal stability compared with the IMU-based approach, achieving an RMSE of 2.76° while reducing drift and measurement jitter. Phantom-based reconstruction experiments further demonstrate consistent volumetric visualization across multiple scanning trajectories and inclusion sizes. Statistical analysis indicated that reconstruction accuracy was significantly influenced by both inclusion diameter and scanning trajectory, with a significant interaction between the two factors. The proposed framework successfully reconstructed the inclusions under all tested acquisition patterns, suggesting feasibility across different scanning conditions; however, reconstruction accuracy depended on both target size and scanning trajectory.

The results support the hypothesis that breast ultrasound scanning, which is largely constrained to surface-following motion with limited angular variation, can be effectively represented using a reduced-dimensional pose model. By aligning the sensing strategy with the intrinsic motion characteristics of breast scanning, the proposed framework reduces hardware complexity while preserving the spatial consistency required for volumetric reconstruction.

Nevertheless, several limitations remain. The present study was evaluated using phantom-based experiments under controlled scanning conditions, and segmentation of reconstructed inclusions was performed semi-automatically. Furthermore, the reduced 2DoF representation may become less accurate under complex out-of-plane probe motion, substantial tissue deformation, or highly irregular breast geometries encountered in clinical practice. Future work will therefore focus on clinical validation using patient data, automated segmentation and reconstruction optimization, multi-observer reproducibility analysis, and extension of the sensing framework to accommodate more complex scanning dynamics.

Overall, the proposed approach demonstrates the feasibility of mechanically simplified pose sensing for freehand 3D ultrasound reconstruction and may support future development of compact point-of-care breast imaging systems for volumetric visualization and longitudinal lesion monitoring.

## Figures and Tables

**Table 1:** Summary of 3D ultrasound approaches for breast cancer screening.

Methods	Studies	Key Features	Advantages	Limitations
HHUS	[9,10,11]	Enhanced transducers, real-time imaging	Widely available, cost-effective	Operator-dependent, lacks volumetric data
ABUS	[12,13,14,15]	Motorized probe movement, 3D data acquisition	Reproducible, improved accuracy in dense tissue	Expensive, limited portability
FWET	[16,17]	Additional data with tracking system	Compatible with existing probes	Complex setup, environment-sensitive
ARABS	[18]	Actuator-driven probe, marker-based navigation	Standardized scanning, minimal user error	Bulky, requires mechanical integration
AI-3DUR	[19,20,21,22]	Deep learning for pose estimation and reconstruction	Real-time capability, minimal hardware	Needs training data, model generalizability

**Table 2:** Summary of volumetric reconstruction for phantom inclusions across object sizes and scanning trajectories (*V*_1~_*V*_4_).

Diameter (mm)	Scanning Trajectory	Absolute Error (mm^3^) (Mean ± Std. Dev)			Relative Error (%) (Mean ± Std. Dev)		
5	*V*_1_	11.12	±	4.59	16.99	±	7.01
5	*V*_2_	15.70	±	8.31	23.99	±	12.70
5	*V*_3_	8.89	±	5.87	13.58	±	8.96
5	*V*_4_	21.50	±	10.19	32.85	±	15.57
7	*V*_1_	9.54	±	8.77	5.31	±	4.88
7	*V*_2_	42.15	±	21.35	23.47	±	11.89
7	*V*_3_	30.67	±	13.42	17.08	±	7.47
7	*V*_4_	39.99	±	22.73	22.27	±	12.66
10	*V*_1_	74.08	±	49.92	14.15	±	9.53
10	*V*_2_	71.63	±	63.17	13.68	±	12.06
10	*V*_3_	94.56	±	34.14	18.06	±	6.52
10	*V*_4_	103.14	±	52.57	19.70	±	10.04

**Table 3:** Shapiro–Wilk and Levene’s test results for the two-way ANOVA model.

Test	Statistic	*p*-Value
Shapiro–Wilk (residuals)	W = 0.992	0.732
Scanning Trajectory	F = 1.761	0.069

**Table 4:** Results of the two-way ANOVA evaluating the effects of object diameter and scanning trajectory on volumetric reconstruction error.

Factor	*df*	*F*	*p*-Value
Inclusion Diameter	2	3.30	0.041 **
Scanning Trajectory	3	8.37	<0.001 ***
Diameter × Trajectory	6	2.75	0.016 **

**: *p* < 0.05, ***: *p* < 0.001.

**Table 5:** Post hoc Tukey HSD comparisons showing pairwise differences in relative reconstruction error among scanning trajectories within each inclusion diameter.

	Comparison (Diameter)	Mean Difference (%)	*p*-Value
5 mm	V1 vs. V2	7.02	0.532
	V1 vs. V3	−3.38	0.913
	V1 vs. V4	15.85	0.020 **
	V2 vs. V3	−10.4	0.202
	V2 vs. V4	8.83	0.334
	V3 vs. V4	19.23	0.004 **
7 mm	V1 vs. V2	18.15	<0.001 ***
	V1 vs. V3	11.74	0.05
	V1 vs. V4	16.93	0.002 **
	V2 vs. V3	−6.41	0.466
	V2 vs. V4	−1.22	0.992
	V3 vs. V4	5.19	0.637
10 mm	V1 vs. V2	−0.47	1
	V1 vs. V3	3.9	0.807
	V1 vs. V4	5.55	0.585
	V2 vs. V3	4.37	0.748
	V2 vs. V4	6.02	0.518
	V3 vs. V4	1.65	0.981

** *p* < 0.05; *** *p* < 0.001.

## Data Availability

The data presented in this study are available on request from the corresponding author.

## Abbreviations

The following abbreviations are used in this manuscript:

3D	Three-Dimensional
2DoF	Two Degrees of Freedom
6DoF	Six Degrees of Freedom
ABUS	Automated Breast Ultrasound Systems
AE	Absolute Error
AI-3DUR	Ai-Assisted 3d Ultrasound Reconstruction
ANOVA	Analysis Of Variance
ARABS	Autonomous Robotic And Actuator-Based Systems
BI-RADS	Breast Imaging-Reporting And Data System
CNN	Convolutional Neural Network
CNR	Contrast-To-Noise Ratio
FFDM	Full-Field Digital Mammography
FWET	Freehand Ultrasound With External Tracking
HHUS	Handheld Two-Dimensional Ultrasound
HSD	Honestly Significant Difference
ICDR	Incremental Cancer Detection Rate
IMU	Inertial Measurement Unit
NRRD	Nearly Raw Raster Data
PPV	Predictive Value
RE	Relative Error
RMSE	Root Mean Square Error
UST	Ultrasound Tomography
